# Spatial limitations in averaging social cues

**DOI:** 10.1038/srep32210

**Published:** 2016-08-30

**Authors:** Joseph Florey, Colin W. G. Clifford, Steven Dakin, Isabelle Mareschal

**Affiliations:** 1 Department of Experimental Psychology, Queen Mary University of London, Mile End Rd, London, UK; 2 School of Psychology, UNSW Australia, Sydney, Australia; 3 Optometry & Vision Science, University of Auckland, Auckland, New Zealand

## Abstract

The direction of social attention from groups provides stronger cueing than from an individual. It has previously been shown that both basic visual features such as size or orientation and more complex features such as face emotion and identity can be averaged across multiple elements. Here we used an equivalent noise procedure to compare observers’ ability to average social cues with their averaging of a non-social cue. Estimates of observers’ *internal noise* (uncertainty associated with processing any individual) and *sample-size* (the effective number of gaze-directions pooled) were derived by fitting equivalent noise functions to discrimination thresholds. We also used reverse correlation analysis to estimate the spatial distribution of samples used by participants. Averaging of head-rotation and cone-rotation was less noisy and more efficient than averaging of gaze direction, though presenting only the eye region of faces at a larger size improved gaze averaging performance. The reverse correlation analysis revealed greater sampling areas for head rotation compared to gaze. We attribute these differences in averaging between gaze and head cues to poorer visual processing of faces in the periphery. The similarity between head and cone averaging are examined within the framework of a general mechanism for averaging of object rotation.

## Introduction

### Social Interactions Using Gaze

The ability to determine where someone’s attention is directed is a critical part of human interaction and communication^1^,^2^. Information about gaze direction and head rotation are key non-verbal cues that we rely on to determine another’s focus of attention^3^. These cues can be differentiated with high precision^4^,^5^,^6^ and are processed with specialised neural mechanisms^7^,^8^. Although previous research has largely focussed on how these cues are interpreted when presented in isolation, recently there has been increased interest in how they can be rapidly averaged within a group, sometimes referred to as *ensemble coding*^9^.

The perception of gaze direction is critical to human social interactions. It influences the processing of emotion^10^, can cue shifts in attention^11^,^12^ and is reportedly abnormal in individuals with conditions such as autism spectrum disorder^13^,^14^. Interestingly it has been found that gaze direction is a stronger cue to attention when the cueing comes from a group of people rather than an individual. For example, Gallup *et al*.^15^ found that when a participant walked past a group of live actors, the greater the number of people in a crowd performing the same behaviour (e.g. looking upwards), the more likely an individual was to look at the location cued by the group^15^,^16^.

Perceived direction of gaze is derived from information about the eyes (position of the iris within the sclera e.g.^17^) and the rotation of the head^4^,^18^, with both cues combined to produce an overall percept of gaze direction^19^. Although precision of judgements of gaze direction is generally high when faces are viewed foveally, performance decreases when faces are in the periphery^20^,^21^. In particular Florey *et al*.^22^ found that in the periphery, although observers could still discriminate between leftwards and rightwards gaze directions, perceived direction of gaze was more influenced by head rotation. Since there is a dedicated system of neurons for processing gaze direction and head rotation located in the superior temporal sulcus^7^,^8^ it may be that the pooling of multiple sources of these cues (e.g. making a judgement about a crowd) also engages specialised mechanisms. That is, there may be a specific mechanism for pooling gaze direction which pools outputs from these face specific regions. This would be consistent with research^23^ where the authors compared correlations between participants’ mean error for face averaging tasks with more basic tasks such as orientation and colour averaging. They found that participants’ error for the two face tasks was correlated although neither task was correlated with the low level tasks. The authors concluded that there was no generic mechanism for averaging visual information (e.g. colour, orientation, faces, etc). However, given the diversity of their averaging tasks, a generic mechanism may still exist which averages *directional* cues, regardless of whether they arise from social (faces) or non-social sources. Here we examine whether observers’ ability to average the direction of cues of different modalities (rotated heads and rotated cone stimuli) is any different to averaging those of the same modality. If a generic rotation pooling mechanism exists then performance should be the same whether all the elements are from the same modality or not.

### Judgements about groups: Equivalent Noise and Reverse Correlation

How do we rapidly extract information about the world? One suggestion is that the visual system extracts *summary statistics* (e.g. mean or variance) of the visual information around us. For example, when perceiving the leaves of a tree, we represent information about the average size, shape and colour rather than information about every leaf^24^,^25^. This has been shown for basic visual properties such as size^26^ or orientation^27^. For example, Parkes *et al*.^27^ presented groups of oriented Gabor patterns to participants in their periphery and asked them to make judgments about the orientation of these elements. They found that participants could not accurately report the orientation of any individual Gabor, despite this; they were able to report the average orientation of the patterns in the array above chance. They proposed a form of “compulsory averaging” of visual information in the periphery and suggested that, in peripherally viewed cluttered displays, people cannot extract information about the individual elements.

In order to measure what limits observers’ averaging of information, equivalent noise methods have been developed. Barlow^28^ proposed that the effect of neural noise on detection performance could be treated as being the same as light (going so far as to call such noise, dark light). One could then quantify the amount of light-noise one had to add to the stimulus to match the effect of dark noise. This amount of light is equivalent noise and has since been used to great effect to psychophysically quantify the limits of vision. Notably, Pelli^29^ used it to show that noise associated with photon-absorbance, and not neural noise, limits detection performance under many circumstances, indicating that the visual system is limited by its “front-end” and not later neural processing. Dakin^30^ showed that the paradigm used for quantifying EN could be adapted to study limits on local and global visual processing. He found that by quantifying limits on observers’ ability to make judgements of averages (now called ensemble processing) as a function of external noise (signal variability) one could use the same approach to derive equivalent noise and efficiency in the setting of texture perception^25^.

When applied to averaging (e.g. orientation, motion and size^30^,^31^,^32^) this technique assumes that there are two limits on an observer’s ability to extract average information about multiple samples; their *internal noise* and their *effective sample-size*. The internal noise refers to the uncertainty associated with processing any individual element in an array (e.g. how accurate we are at judging the gaze direction of a single face). Sample size refers to the number of elements from the array that the observer’s seem to be combining when estimating the average (e.g. how many faces from a crowd are used to make a judgement). This measure of effective sample size tells us how many samples an ideal observer would have to be using in order to obtain the observed thresholds given constant internal noise. This does not necessarily mean that on *every trial* a participant with a sample size of *n* is using *n* elements in their mean estimate, but rather gives us a measure of how efficiently they are combining information across the array. By considering these two separate limits on averaging performance, we are able to show when averaging performance improves, and whether this results from reduced internal noise or an increased effective sample size.

**Figure 1:**
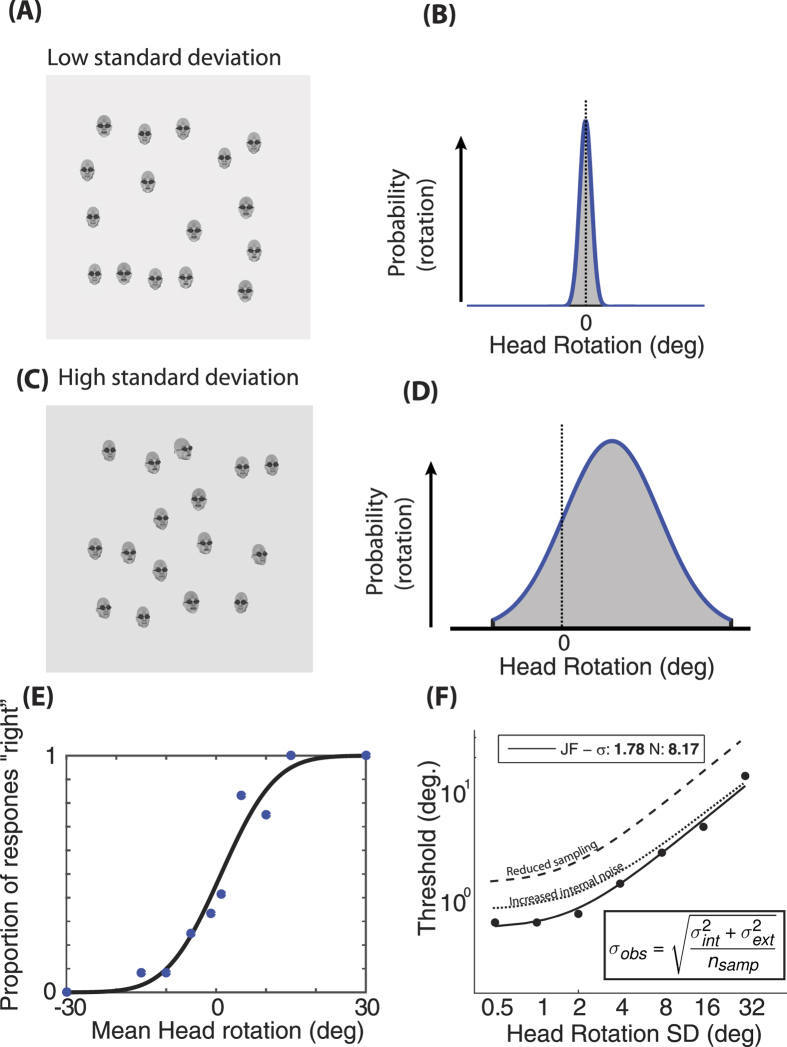
(**A**–**D**) Examples of stimuli and the distributions they are drawn from. (**A**,**B**) Low external noise and a mean head rotation close to direct. (**C**,**D**) High external noise and a rightwards mean head rotation. (**E**) An example of performance on a head rotation task for one observer (JF). Circles show the proportion of times participants responded “right” for each mean head rotation presented. The solid black line shows the best fitting cumulative Gaussian function fit to these points based on maximum likelihood fitting. Discrimination threshold is taken as the standard deviation of the Gaussian function for this data set. (**F**) An equivalent noise function for one participant. Reduced effective sample size is characterised by an upward shift in the function, whereas increased internal noise is characterised by an increase in thresholds at lower levels of external noise, but approaching the same asymptote at high levels of external noise. Face stimuli were created using FaceGen Modeller 3.5 (facegen.com) and Poser 10 (my.smithmicro.com/poser-3d-animation-software.html).

Determining these limits on performance cannot be achieved by looking at changes in sensitivity alone. These two measures of performance can be estimated by measuring changes in observers’ discrimination thresholds (in this case the smallest shifts in mean head rotation that observers can discriminate between left and right Fig. 1e) as a function of increasing external noise (in this case changes in the *variability* of head rotation). When external noise is low (i.e. all faces in an array are facing roughly the same direction Fig. 1a,b) discrimination is generally good and limited predominantly by the observer’s internal noise. When external noise is high (faces are all looking in a wider-range of directions Fig. 1c,d), discrimination performance is poor and predominantly limited by how many faces are combined to estimate an average (since the internal noise on any one estimate is now swamped by the external noise). The more faces that are pooled the better the performance. Estimates of an individuals’ sample size (*n*_*samp*_) and internal noise (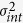) can be extracted by plotting their discrimination threshold (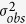) as a function of the standard deviation of the directions present in the stimuli (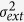) and fitting an equivalent noise function (Fig. 1f) to the data.

Using this method, it has been shown that participants use only a subset of samples in an array of Gabor patches to estimate the average orientation^30^. Estimates of *subsampling* vary across the literature and depend on the total number of samples in the array, the stimulus property being averaged as well as stimulus presentation duration^33^. Estimates range from as many as 80 samples in densely populated arrays of 1024 oriented Gabors^30^ to as low as just 1 out of 100 for motion direction integration in young children^34^. Using a different method, Sweeny and Whitney^9^ found that the variance in observers’ perceived mean gaze deviation of a group of faces decreased when more faces were added to the group (up to four faces). Although their method demonstrates that participants must be averaging to some extent, it does not indicate how many samples (if the population size had been larger) are used. Here we will use the equivalent noise paradigm to examine how efficiently gaze direction and head rotation can be averaged in crowds, a critical first step to understanding how good we are at making (social) judgments about a group.

It is important to note that this procedure is agnostic as to the underlying mechanism. Equivalent noise allows us to characterise observers’ performance in terms of how many samples they are *effectively* averaging. It does not tell us what mechanism allows them to produce this behaviour. Another shortcoming of equivalent noise methods is that they do not inform us as to *which* samples are pooled in the array when an observer judges the average. For example we might expect that observers rely more heavily on samples falling close to fixation where resolution is high, but this need not be the case. Reverse correlation methods provide a means of examining this and have been used in a variety of psychophysics tasks^35^,^36^,^37^,^38^,^39^. In conventional reverse correlation, noise patterns are added to a stimulus and the observer is required to perform a discrimination task. The observer’s performance is then correlated with the noise pattern, on a trial-by-trial basis, to determine which noise patterns improved, and which hindered, performance. By adding all the noise patterns that improved (i.e. were positively correlated with) performance and those that hindered (i.e. were negatively correlated with) performance, it is possible to create a map that indicates which parts of the stimulus were used to perform the task.

### Averaging of social (faces) stimuli

Although people’s ability to average basic visual properties has been well studied, it is useful to expand these methods to broader forms of information. For example, Haberman and Whitney^40^ examined whether participants could average information about facial emotions. They found that when an array of faces with different emotional expressions was briefly presented, participants were able to accurately report the average emotion of the array, suggesting that the ability to infer summary statistics is not limited to basic visual properties. Interestingly, when participants were tested on their memory of the individual faces in the arrays, they only remembered one, suggesting that calculating the average also did not require having conscious representations of each face. The authors suggest that this “ensemble coding” is a rapid process that allows us to quickly extract the gist information from a scene. More recently, Sweeny and Whitney^9^ presented sets of up to four faces around a fixation point and asked participants to report the average gaze direction. Variance in participants’ responses decreased when more heads (up to 4) were viewed, suggesting that observers were able to pool gaze directions over space. Importantly, the gaze direction of these faces was produced as a result of the Wollaston illusion^41^. In this illusion, the gaze direction of the eyes in a face is biased by the rotation of the surrounding facial features; this allows the perceived gaze deviation to be manipulated without changing the properties of the eyes themselves. This suggests that the pooling process required higher level processing beyond simply extracting edges or line orientation (e.g. of the iris in the sclera).

Here we measure the limits of averaging for both gaze direction and head rotation in order to determine whether the mechanism(s) for *averaging* are unique to faces or more generic. Given that gaze deviation is more difficult to resolve than head rotation, particularly for non -foveally viewed faces, we expect that head rotation will be more efficiently averaged (since some heads will necessarily be presented in the observer’s periphery). We will also examine whether there is a generic mechanism that pools information, regardless of the object class (using social and non-social stimuli). Finally, we will apply a reverse correlation technique to estimate regions of integration: locations in the stimulus array that underlie observers’ performance. The reverse correlation maps will also allow direct comparison between sampling efficiencies in the different tasks and stimulus regions of integration (e.g. a smaller highlighted area for conditions with lower effective sample size).

## Results

### Equivalent noise (EN) results

**Figure 2:**
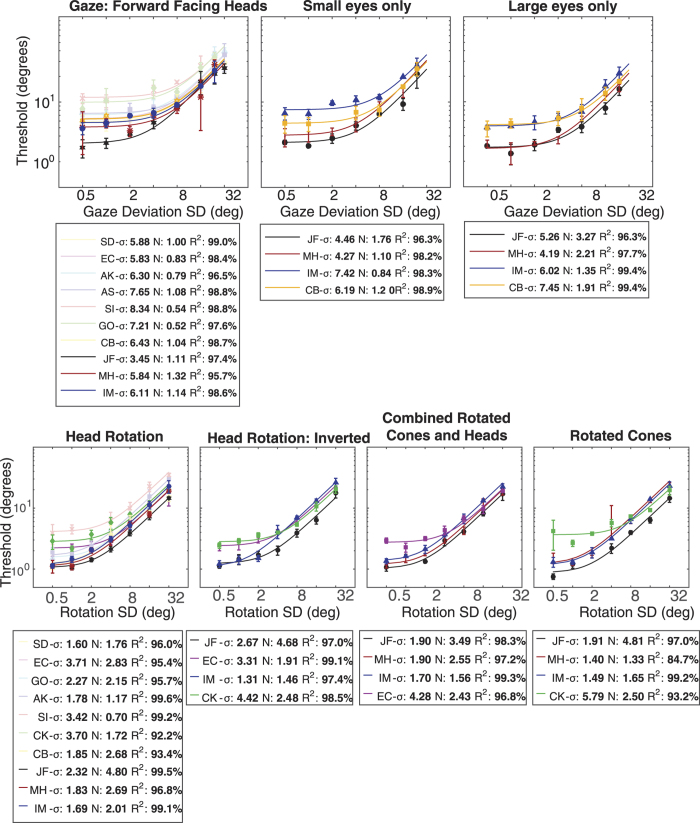
Equivalent noise functions (lines) fit to the average gaze direction discrimination threshold (symbols, error bars represent +/−1 SEM) for each participant in all conditions. Top (left to right): the three gaze conditions; full faces, eyes only and large eyes only. Bottom (left to right): upright heads, inverted heads, combined head and cone stimuli and cone only stimuli. R^2^ goodness of fit parameters and estimates of internal noise and effective sample size are below each plot. For clarity, data from participants who performed only one of the conditions are semi-transparent.

Figure 2 shows the EN function fits to the data for each observer and stimulus type with their corresponding internal noise and effective sample sizes.

To compare the two main stimulus conditions (gaze deviation and head rotation in upright faces), independent samples t-tests were carried out on the two equivalent noise parameters. The averaging of head rotation was associated with greater sample sizes (M = 2.31) than gaze direction (M = 0.94, *t*(9.97) = 3.6 *p* < 0.001) and averaging of head rotation was associated with less internal noise (M = 2.27°) than gaze direction (M = 6.15°, *t*(18) = 7.83 *p* < 0.001). Taken together these results show that participants were able to average the rotation of heads and cones more efficiently (i.e. using more samples) and with less internal noise than average gaze direction.

Two 1 × 3 random effects ANOVAs were carried out on the three gaze deviation conditions: full face, eyes only and large eyes only. No significant differences were found between the internal noise estimates for each stimulus type (*F*(2, 15) = 0.515 *p* = 0.61). A significant main effect was found for the effective sample size of the three conditions (*F*(2, 15) = 9.99 *p* = 0.002). Post-Hoc Bonferroni corrected t-tests revealed that the “large eyes only” condition was associated with a greater effective sample size (M = 2.2) than the main gaze condition (M = 0.94). Similar 1 × 4 ANOVAs were carried out on the four rotation conditions: Head rotation, inverted head rotation, cone rotation and the mixed cone and head. No significant difference was found between the four conditions for either internal noise (*F*(3, 18) = 0.169 *p* = 0.92) or effective sample size (*F*(3, 18) = 0.132 *p* = 0.94). These results suggest that for both the head rotation and gaze deviation control stimuli, the internal noise associated with processing any item was consistent regardless of the manipulation. Increasing the size of the eye-region and removing the head surround caused observers to sample more efficiently from the array of gaze deviation stimuli. None of the rotation stimuli controls caused any change in observers’ effective sample size.

**Figure 3:**
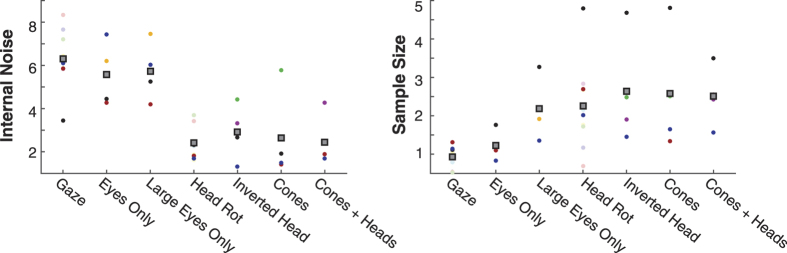
Summary of equivalent noise parameters across all conditions. Coloured circles show data from each participant (corresponding to the colours in Fig. 4). Grey squares show the mean across participants.

Figure 3 summarises the data for all the stimulus conditions. The average internal noise falls into two groups, with the three gaze deviation conditions associated with higher levels of internal noise than the head/cone rotation conditions. Sample size is approximately 1 for both the main gaze condition and the “eyes only” condition suggesting that there is little to no averaging occurring for these stimuli. In contrast, the head, cone, and mixed conditions as well as the “large eyes” condition all have mean sample sizes greater than 2 suggesting some averaging.

### Mixed stimulus averaging

We found no difference in performance between averaging stimuli of the same type and a mixture of stimulus type (heads and cones). In order to verify that participants were not simply ignoring one type of stimulus (e.g. only using the heads in the mixed condition), we collected additional data on 4 participants for the two highest noise conditions with stimuli made up of both four heads and sets of 2 heads and 2 cones. The total area of the stimulus was reduced in the smaller arrays so that the density of the items was maintained between the first and second experiment. If participants were only averaging across stimulus type we would expect thresholds to increase as the number of samples of the given stimulus type decreased. However, if they were averaging across samples regardless of the stimulus type, then we would expect to see no difference between conditions. Only high external noise conditions that require maximum pooling were tested.

**Figure 4:**
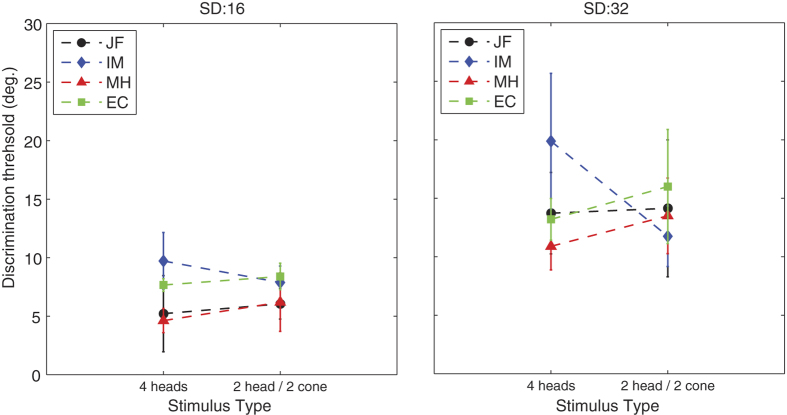
Mean rotation discrimination thresholds for sets of 4 heads and sets of 2 heads and 2 cones. Each line shows data for a separate participant (error bars are +/− one standard deviation) and each graph shows data for a single external noise level.

The results from these two conditions are plotted in Fig. 4. Paired sample t-tests showed no differences in discrimination thresholds between the two stimulus types (16: *t*(3) = 0.434, *p* = 0.694; 32: *t*(3) = 0.224, *p* = 0.837).

### Reverse Correlation

To generate reverse correlation maps for each participant, the location and rotation of each face in the stimulus was stored along with the participants’ response (leftwards or rightwards). Only the trials where the mean direction was 0° were used for the analysis (90% of total).

**Figure 5:**
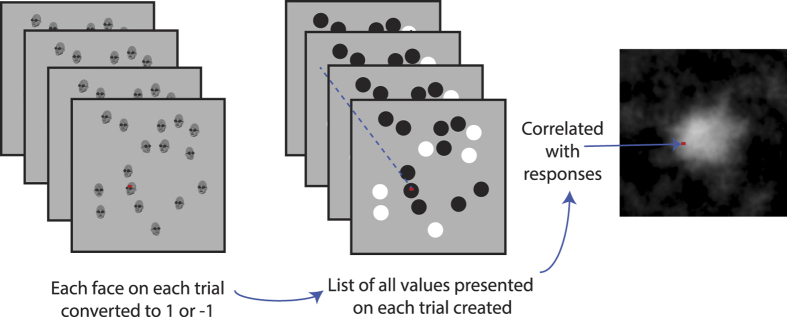
Reverse correlation method. On every trial, pixels in the stimulus that were part of a face were assigned a value of [−1 = black] or [1 = white] indicative of the gaze deviation (or head rotation) of the face stimulus at that location (pixels in areas of the stimulus without a face were not assigned a value). At the end of *N* trials, the pixels in the *N* stimuli are correlated with the *N* responses given. Each pixel therefore represents a correlation value, whose magnitude is an index of the influence the faces had on the observer’s decision. A reverse correlation map is constructed (rightmost) whereby the influence of a given location in the stimulus is represented by a grey scale.

**Figure 6:**
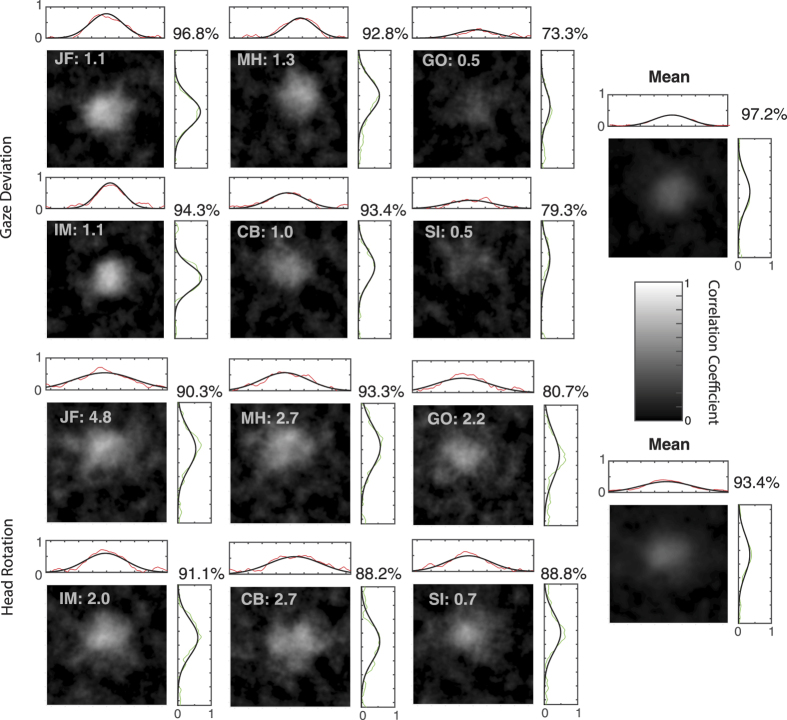
Reverse correlation images for each participant and the mean across participants for both gaze deviation and head rotation judgements. Grey text in the top left of each plot shows the participant and their sampling efficiency from the corresponding condition in the main experiment. Above and right of each image are the 2D Gaussian fits (black lines) to the smoothed pixel correlation values (red and green points). Percent variance accounted for by fit is shown in the top right of each plot.

A reverse correlation map was generated for each participant (Fig. 5) by creating an array the size of the total stimulus area for each stimulus trial. All pixels that contained a face within the stimulus trial were assigned a value according to the orientation of the face at that location (1 if rightwards oriented and −1 if leftwards oriented). Pixels in stimulus regions that did not contain a face were not assigned a value. For every trial, we obtained a set of pixel values that were then correlated with the participants’ response for the trial. This results in a reverse correlation map where each pixel’s value is the correlation coefficient for that pixel across all responses and trials. These images were then smoothed with a Gaussian filter with a standard deviation of three pixels. These images are shown in Fig. 6 along with the mean of the six participants.

Two dimensional Gaussian functions were fit to the smoothed maps (Fig. 6). The width parameter of the 2D Gaussian was greater in the head rotation conditions for both the abscissa (*t*(10) = 3.73, *p* < 0.01) and ordinate (*t*(10) = 3.71, *p* < 0.01) directions, suggesting that participants were averaging over a greater area in the head condition relative to the gaze, consistent with the sample size results. There was no significant correlation between the mean width of the two gaussians for each participant and their effective sample size for gaze deviation (*r* = −0.35, *p* = 0.49) or head rotation (*r* = 0.67, *p* = 0.15).

## General Discussion

We sought to investigate how people make rapid estimates about the average gaze and head rotation in a group of faces. Using an equivalent noise method, we estimated the internal noise and sampling efficiencies associated with pooling these types of cues. In addition, a reverse correlation analysis was applied to determine which elements in the array were being used to perform the task.

The key findings from this study are as follows: **(1)** pooling of gaze deviation is severely limited by size and proximity to fixation since number of samples used in the main experiment is approximately 1, however increasing the size of the eyes (presented alone) improved averaging. **(2)** Pooling of head rotation is more efficient than gaze with observers using approximately 2–4 samples. **(3)** Rotation cues from non-social cone stimuli were averaged as well as heads when matched in size. We also report that observers are able to combine information from head and cone directions. **(4)** Reverse correlation analysis revealed that there was a general trend to preferentially use samples in the centre of an array. In addition, participants used samples from a significantly greater area in the head rotation condition compared to gaze, concomitant with the increase in effective sample size between the conditions. Surprisingly however, no clear relationship emerged between the efficiency of sampling of individual participants and the reverse correlation maps, which may result from trial by trial fluctuations in strategies.

The finding of inefficient pooling of gaze deviation is mainly consistent with previous findings. Our results for effective sample size are lower than the majority of studies reporting samples for averaging size^33^ and motion^31^, however Manning *et al*.^34^ have observed sampling efficiencies at or below one for motion in children. Given that judging gaze direction of faces in the periphery is much poorer than in the fovea^20^,^22^, it is not surprising that averaging gaze direction in a crowd of faces (most of which fall in the periphery) is also poor. The increase in effective sample size in the “large eyes” condition further supports this explanation of reduced peripheral resolution for the gaze condition. Note however that although we report that increasing the eye size brought the effective sample size to a similar level for gaze averaging as for the head averaging condition, this does not reflect averaging performance under naturalistic conditions. Indeed, eyes are always embedded in a (larger) head and therefore heads and eyes will never be matched in size.

Sweeny and Whitney^9^ reported that participants were able to average gaze deviation over multiple faces, though they make no claims about the number of faces being used. This appears inconsistent with our result that participants are not using more than a single sample when averaging gaze deviation. Crucially, their faces did not actually vary in the offset of the pupils; instead they varied the orientation of the features around the eyes, producing shifts in perceived gaze direction as a result of the Wollaston illusion. This may be why their results suggest that up to four faces are being used in some cases, since the important cues (e.g. the orientation of the features around the eyes and overall head rotation), are easier to resolve in the periphery than the eyes themselves and therefore can be used in the averaging process more efficiently.

We find that head rotation was slightly more efficiently averaged than gaze deviation, with the estimated number of samples greater than 2. This approximates earlier reports^30^,^31^ that suggest that the number of samples a participant uses is equal to the square root of the total number. This improvement in effective sample size is most likely the result of head rotation being more easily discriminated in the periphery^20^. Inverting the heads did not have a significant effect on effective sample size nor did averaging arrays of cones, or creating arrays that contained both types of stimuli. Given that it is unlikely that we have a specific mechanism to process cone direction, a more parsimonious account would be the involvement of a generic mechanism that averages the rotation of objects.

It may be surprising that samples from different stimulus categories (heads and cones) are pooled as well as samples from the same category. Since summary statistics are believed to allow the rapid extraction of “gist” from a complex environment it is not surprising that they could be used to extract the average head rotation of a crowd. It is less intuitive for unrelated objects to be pooled together. Chong and Triesman^42^ have demonstrated that the average of multiple perceptual groups can be extracted at the same time. Participants were able to simultaneously perceive the average size of groups of circles of different colours, though It has been suggested that this process comes with a cost to accuracy^43^. It may be that in our case the two types of stimuli are averaged separately and then combined to create a global average. A parsimonious account of our data involves a generic averaging mechanism, however this need not be a high level mechanism dedicated to rotation per se. For example, preliminary evidence suggests that observers are able to average 2D position^44^ cues (offsets in spatial position of features that arise as a consequence of 3D rotation). It remains to be seen whether these 2D positional cues are used in our study or whether observers are engaging a specific mechanism for averaging 3D rotation.

The reverse correlation analysis revealed that on average, participants use the central elements in a stimulus to make their decision. Although there was no fixation point it would make sense for participants to fixate centrally as this would be most efficient for processing and is consistent with observers’ bias to fixate in the centre of a screen^45^,^46^,^47^. Prima facie, this result is inconsistent with findings from Wolfe *et al*.^48^ who found that observers performed equally well on an emotion averaging task, regardless of whether information was available around fixation or not. It is possible that this may reflect differences in the stimuli used; peripheral emotion can be perceived with some degree of accuracy^49^ whereas peripheral gaze cannot^21^,^22^. Alternatively, it may be the case that observers alter their sampling strategy to adapt to the structure of the image. For example, they may be biased towards using foveal information when it is available but distribute their attention further when it is not. Alvarez^24^ used simulations to show that a weighted averaging (with greater weights given to elements sampled with greater precision) would yield more accurate performance than an equally weighted average when samples were estimated for a size averaging task. The individual differences in our reverse correlation data do not suggest that sampling strategy aids the averaging process. For example, both JF and IM have the clearest central bias, with similar sampling areas yet have different sampling efficiencies (e.g. 4.8 vs 2.0 for head rotation). Instead, these maps suggest that the differences in effective sample size between participants are more likely due to other factors such as differences in the way they compute the average (e.g. using a weighted average or taking the maximum of a subset).

The direction of attention of a crowd can be an even stronger cue for attention than that of an individual^15^ so it makes sense that there might exist a mechanism for rapidly pooling this information. Our results suggest that it is likely that head rotation is rapidly pooled as this can be done more efficiently than gaze. It is possible that after an initial summary statistic is extracted for head rotation, the individual gaze deviations of the members of the crowd may be scanned in a serial fashion to get a more accurate estimate of the true point of interest.

## General Methods

The equivalent noise paradigm was used to estimate internal noise and sample-size for pooling gaze direction and head rotation in groups of faces. The data were fit using an equivalent noise function (equation 1) to each individual’s discrimination thresholds across a range of (gaze or head) external noise levels.


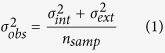


Where σ_*obs*_ is the observer’s discrimination threshold, 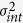 their internal noise, 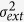 the added external noise and *n*_*samp*_ the effective number of samples used to estimate the mean (e.g. Fig. 1).

### Estimating thresholds

The observer’s task was to categorise the mean direction of gaze (head or cone rotation) as either to their left or to their right. Thresholds were measured for each level of external noise using a method of constant stimuli (MOCS). Ten mean gaze deviations were presented 12 times each resulting in 120 (randomly ordered) trials per run. The proportion of times the participant responded “rightwards” were plotted against the mean gaze deviation and a cumulative Gaussian function fit to the data using a maximum likelihood estimate method (Fig. 1e). Observers completed 3 runs per level of external noise, and thresholds were averaged across the three estimates.

### Participants

Participants were two authors, JF and IM and nine naïve undergraduates at Queen Mary University of London. All participants had normal or corrected to normal vision. Not all participants completed all conditions (each participant is allocated a unique set of initials on data figures). All participants gave informed consent and methods were approved by and carried out in accordance with guidelines from the University’s ethics board.

### Stimuli

Stimuli were sets of sixteen individual faces with each face subtending approximately 2 × 2 degrees of visual angle. On each trial, these were randomly positioned within an 18 × 18 degree square in the centre of a uniform grey screen with no overlap between stimuli. The identity of each face was chosen at random from a set of four identities, two male and two female made with Facegen (Singular Inversions 2016).

In the crowd stimulus, each face’s gaze deviation (head rotation) was drawn from a Gaussian distribution centred on the mean gaze deviation (where negative values indicate leftwards gaze). In most cases, the standard deviations for these distributions were 0.5, 1, 2, 4, 8, 16 and 32 degrees. Due to physical constraints on the gaze stimuli (gaze deviation values that occasionally exceeded the possible range for the human eye), a maximum standard deviation value of 24 degrees was used for all gaze discrimination conditions. The mean rotation values for the MOCS were fixed across observers but changed depending on the stimuli and task difficulty so that a psychometric function could always be fit to the data.

#### Gaze deviation stimuli

Faces for the gaze deviation conditions were created by first generating greyscale head stimuli with neutral expressions using FaceGen software. The eyes were then removed and the face stimuli were exported to Matlab where new greyscale eye stimuli that allowed for very fine control of gaze deviations were inserted into the face. The pupil and iris were made the same dark grey colour so that the gaze deviation was clearly visible.

**Figure 7:**
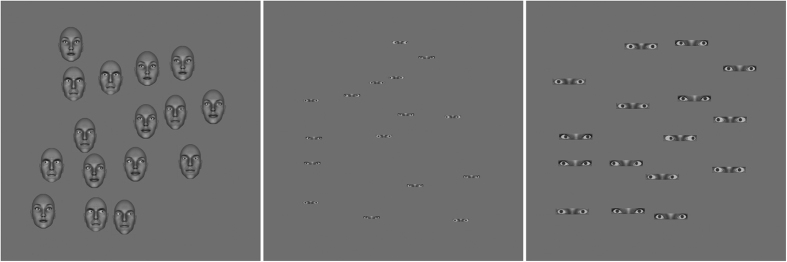
Examples of the arrays of 16 gaze deviation stimuli used. From left to right: the “full head” condition, the “eyes only” condition and the (enlarged) “eyes only” condition. Here all stimuli have an external noise value of 16°. Face stimuli were created using FaceGen Modeller 3.5 (facegen.com) and MatLab R2015a (uk.mathworks.com/products/matlab).

There were three different stimulus conditions for the gaze judgement task; one with the whole head present, one with only the eye region visible and one where the eye region was enlarged to approximately match the total area of the heads in the head rotation conditions (Fig. 7).

For low levels of external noise, gaze deviations were drawn from a Gaussian distribution whose mean was centred on one of the following [−15°, −9°, −6°, −3°, −1°, 1°, 3°, 6°, 9°, 15°], and standard deviation determined by the noise level (SD = 0.5; 1; 2; 4 and 8). For high levels of external noise, the gaze deviations were drawn from a Gaussian distribution with a mean centred on [−20°, −15°, −10°, −5°, −1°, 1°, 5°, 10°, 15°, 20°] and a standard deviation of either (16, 24).

#### Head rotation stimuli

To generate the full range of head rotations, the original (forward facing) heads created in Facegen were uploaded to Poser software (Smith Micro 2016), which allowed us to rotate the heads in 3D space along a fixed x-axis. By saving each frame from an animation of the head rotating, stimuli could be exported for head rotations that spanned 180° (leftwards to to rightwards) in steps of 0.1°. Black glasses were added to the original Facegen stimuli to remove gaze information.

**Figure 8:**
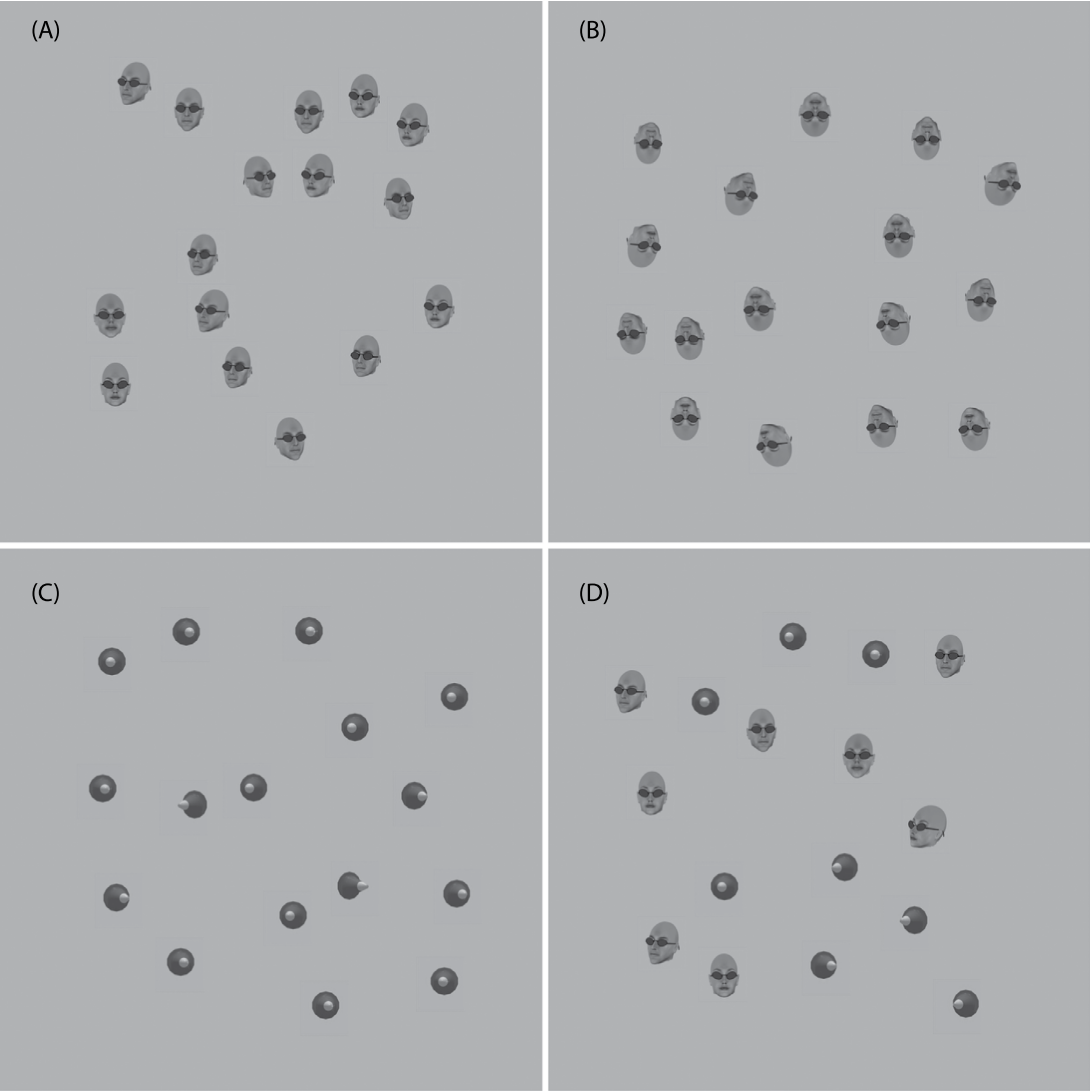
Examples of the four types of stimuli used to probe judgement of mean object rotation. (**A**–**D**) Upright rotated heads, inverted heads, 3D cones and a mixture of heads and cones. All arrays have an external noise of 16°. Face stimuli were created using FaceGen Modeller 3.5 (facegen.com) and Poser 10 (my.smithmicro.com/poser-3d-animation-software.html).

Observers were required to judge the mean direction of head rotation using the same task. For noise SD values from 0.5° to 4° mean head rotations were [−6°, −3°, −2°, −1°, −0.5°, 0.5°, 1°, 2°, 3°, 6°], when the noise SD was 8° and above, the mean values were [−30°, −15°, −10°, −5°, −1°, 1°, 5°, 10°, 15°, 30°]. The faces were also randomly arranged and placed on a lighter grey background to increase the contrast of the edges of the faces. Data was collected for both upright rotated heads and inverted heads (Fig. 8).

#### Cone stimuli

Non-social directional stimuli were created using rotated cones whose shape was based on a 3D model of a traffic cone. The cone was imported into blender software and its shape edited to remove some of the base and textured to give it a white tip (Fig. 8). The full range of rotations was produced in the same way as with the head stimuli. The cone stimuli were approximately the same size as the head stimuli (2 × 2 degrees of visual angle). In the mixed averaging condition, hybrid stimuli containing both cones and heads were created. The type of cue for each element in the array was randomly assigned as either a head or a cone to avoid participants only responding to one stimulus type.

### Procedure

Each trial began with a blank grey screen (500 ms) immediately followed by a 300 ms presentation of the stimulus, followed by a return to the grey screen. Brief presentations were used to ensure participants did not make multiple saccades characteristic of serial averaging. Participants made untimed responses. The participant’s task was to indicate, using a key press, whether the average gaze (head/cone rotation) in the array of stimuli was to their left or to their right. The next trial began as soon as the participant had made their response. No feedback was given.

### Reverse correlation

In order to examine which parts of the stimulus contributed to the averaging process we also collected data on the same task in a separate session, optimised for reverse correlation. In the reverse correlation analysis, the most informative trials are when the mean of the stimulus is at threshold as this ensures that participant’s response will be driven solely by the subsample of stimuli they use in the image. For example, if the mean of an array is 30° rightwards, most faces will be turned rightwards and a rightwards response from the observer will not be indicative of which faces they based their decision on. However if the mean is 0° then faces are equally likely to be either leftwards or rightwards. In this case the observers’ responses will be informative as to which faces they used. In this procedure, the majority of the stimuli (90%) were presented with a mean of 0°. In this case, on any given trial subsets of the faces will be biased to contain either more leftwards or rightwards deviated faces and the observers response will correlate with the subset they used for the task. If an observer has a systematic bias for favouring certain locations within the array (e.g. the centre), then this will be revealed by the presence of a “hotspot” in the reverse correlation map. Catch trials (stimuli with a mean of 10° either leftwards or rightwards) were introduced to ensure that participants were not responding arbitrarily. In all cases, the external noise was fixed at an SD of 16°. 6 participants completed 1200 trials, two authors (JF and IM) and four naive participants.

## Additional Information

**How to cite this article**: Florey, J. *et al*. Spatial limitations in averaging social cues. *Sci. Rep.* **6**, 32210; doi: 10.1038/srep32210 (2016).
